# Evaluation of the impact of serogroup C meningococcal disease vaccination program in Brazil and its regions: a population-based study, 2001-2013

**DOI:** 10.1590/0074-02760160173

**Published:** 2017-04

**Authors:** Camile de Moraes, José Cássio de Moraes, Gabriela Drummond Marques da Silva, Elisabeth Carmen Duarte

**Affiliations:** 1 Universidade de Brasília, Faculdade de Medicina, Programa de Pós-Graduação em Medicina Tropical, Brasília, DF, Brasil; 2 Ministério da Saúde, Secretaria de Vigilância em Saúde, Brasília, DF, Brasil; 3 Faculdade de Ciências Médicas da Santa Casa, São Paulo, SP, Brasil; 4 Universidade de Brasília, Faculdade de Ciências da Saúde, Programa de Pós-Graduação em Saúde Coletiva, Brasília, DF, Brasil

**Keywords:** meningococcal disease, Neisseria meningitides, serogroup C conjugate vaccine, surveillance, vaccination impact

## Abstract

**BACKGROUND:**

Meningococcal C conjugate (MenC) vaccine was introduced as part of the Brazilian National Immunisation Program in 2010 for children < 1 year of age.

**OBJECTIVES:**

The study objective was to evaluate the impact of this vaccination strategy.

**METHODS:**

An observational, mixed ecological and analytical study was conducted, based on time series panel data from surveillance records (2001-2013).

**FINDINGS:**

A total of 37,538 of meningococcal disease cases were recorded during the study period. Of these, 19,997 were attributed to serogroup C. A decrease in meningococcal disease serogroup C (MDC) incidence among children aged < 1 year [65.2%; 95% confidence interval (CI): 20.5-84.7%] and 1-4 years (46.9%; 95%CI: 14.6-79.1%) were found in the three years following vaccination introduction. Vaccination impact on the reduction of MDC incidence varied from 83.7% (95%CI: 51.1-100.0%) in the Midwest region to 56.7% (95%CI: 37.4-76.0%) in the Northeast region.

**MAIN CONCLUSIONS:**

Vaccination against MDC in Brazil had a positive impact on the population of children aged < 1 year, across all regions, and on the 1-4 year-old cohort. Nevertheless, in our view there is scope for improving the vaccination strategy adopted in Brazil.

Meningococcal disease (MD) is endemic in Brazil and sporadic outbreaks have been recorded. Meningococcus is the leading cause of bacterial meningitis in Brazil (MS/SVS 2014). Until 2005, serogroup B was the most frequent isolate in invasive MD cases (67%) across all age groups (PAHO 2007). Thereafter, circulation of serogroup C increased, becoming the most predominant and accounting for over 74% of isolates in 2010 (Ibarz-Pavon et al. 2012).

The incidence of MD was very high, approximately 1.6 cases per 100,000 population, with the highest incidence occurring among infants and young children (Sáfadi et al. 2015), as well as recurring outbreaks in different regions (Gorla et al. 2012, Iser et al. 2012). This led to the inclusion of meningococcal C conjugate (MenC) vaccine in the National Immunisation Programme in 2010 (SI-PNI/MS/SVS 2010). The recommended regimen consisted of two doses (at three and five months of age) and one booster (between 12-15 months of age). Infants aged 12-23 months received one dose of the vaccine in the year of implementation of the program. Three Brazilian regions reached the target vaccination coverage (> 95%) in the first year following program implementation (2011). The Northeast region achieved this in 2013; the Northern region achieved a vaccination coverage ranging between 80% and 90% during the study period (SI-PNI/MS/SVS 2010).

The incidence and serogroup circulation of MD varies across the Brazilian regions. Serogroup C became predominant in the following regions: Southeast, since 2002-2003; Midwest, since 2005-2006; Northeast, since 2007-2008; Northern, since 2008-2009; and, more recently, Southern since 2012 (de Moraes 2016).

Although some studies have evaluated the introduction of the vaccine in Brazilian municipalities (Cardoso et al. 2012, Tauil et al. 2014), there are no reported studies assessing the impact of this strategy on a national scale. The objective of this study was, therefore, to evaluate the impact of the vaccination program in Brazil on the MD incidence rate, according to the country’s geographical regions.

## MATERIALS AND METHODS

*Study type* - This is an observational, mixed ecological (aggregated in time and space) (Morgenstern 1995), analytical study, with time series analysis based on panel data (cross-sectional observations at different points in time) (Hsiao 2007).

*Data source* - This study is based on the Notifiable Diseases Information System (Sinan) database that records all MD cases identified by the surveillance system. MD notification is mandatory in Brazil.

The population databases used were the Live Birth Information System (Sinasc) and the population estimates of the Brazilian Institute of Geography and Statistics (IBGE).

*Study population and period* - All confirmed MD cases occurring in Brazil between 2001 and 2013 and reported on the Sinan system were included in the study.

According to Surveillance Guideline (MS/SVS 2014), cases reported to Sinan are classified as meningococcal meningitis, meningococcemia, or meningococcemia combined with meningococcal meningitis. The following diagnostic criteria for MD case confirmation were used: culture, detection of bacterial DNA by polymerase chain reaction (PCR), antigen detection, clinical-epidemiological criteria (case of close contact with a case confirmed by laboratory tests), gram-staining, and clinical criteria (with petechial or purpuric rash). Serogroup was determined only for MD cases confirmed by culture, PCR, antigen detection and clinical-epidemiological criteria. Serogroup was classified as “unidentified” for MD cases confirmed by gram-staining and clinical criteria.

Unidentified serogroup MD cases were distributed according to the proportion of cases with identified serogroups, taking into consideration region of residence, age group, and the trimester of symptom onset. The study population was composed of confirmed cases of MDC, in addition to “unidentified serogroup” cases, attributable to serogroup C, on the basis of proportional distribution.

All analyses were performed using the MDC incidence, with and without unidentified serogroup case redistribution.

*Study variables* - Dependent variables: trimestral incidence: MDC trimestral rates were calculated for each year of the study, by age group (< 1 year; 1-4 years; 5-9 years; ≥ 10 years), and region of residence;

Independent variables: (i) year (historical trend): “year” variable for the period 2001-2013; (ii) trimesters (season): “month” variable (regardless of the year) was aggregated into trimesters: January-March (category = 0, reference), April-June (category = 1), July-September (category = 2) and October-December (category = 3); (iii) vaccine (impact of vaccination): the time series was divided into periods, according to the stages of vaccination implementation, as follows: 2001-2009 = nº vaccination program (pre-implementation, reference period = 0); 2010 = implementation year (category = 1); 2011-2013 = vaccination post-implementation period (category = 2). The latter was subdivided into the following categories: 2 (year 2011), 3 (year 2012), and 4 (year 2013); (iv) region of residence: Northeast, Midwest, Southeast, South, and North; (v) age: < 1 year; 1-4 years; 5-9 years; ≥ 10 years.

*Data analysis* - The generalised least square (GLS) model with an autoregressive (AR) component equal to 1 was used to analyse the time series. The AR1 model included the temporal dependence of the data, taking into account that the rate in a given time t is influenced by the rate in t-1. This assumption seems plausible for MD trimester rate models.

Stages of model estimates were: (i) models separated by age group (< 1 year, 1-4 years, 5-9 years, and ≥ 10 years) across Brazil, using “year”, “trimester”, “region” and “vaccine” as explanatory variables (the latter having five categories), and using the outcome variable as MDC incidence at a specific age; (ii) models were estimated for each of the country’s regions and to ensure statistical power, the “vaccine” variable, with the three categories defined above (0, 1 and 2), was used; (iii) diagnostic of regression models was performed (details are presented below); (iv) models were repeated using MDC incidence (outcome) without unidentified serogroup case redistribution.

The statistical significance level was set at 5% (type I error) in regression models.

Residual diagnostics of the GLS statistical regression models were performed to evaluate homogeneity of variance between the panels; autocorrelation between the panels; and linearity, independence, and normality assumptions. The Shapiro-Wilk W-test for normal data was performed to assess the normality assumption of the residuals, and the data distribution was evaluated using the Quantile-Quantile plot and the Kernel density plot. To clarify the linearity assumption, distribution plots of the residuals (estimated values versus residuals) were evaluated. The study also evaluated whether residuals were free from serial autocorrelation (white-noise) using Q (Sperling & Baum 2001) and B (Bartlett 1955) statistics. Finally, the Akaike information criterion (AIC) and Bayesian information criterion (BIC) values were assessed, with and without panel standard error heteroscedasticity (Akaike 1974, Schwarz 1978). Based on these results, the decision was taken to adopt the model with the best fit (the model with the lowest AIC and BIC). Whenever the model did not fit GLS assumptions, a note of caution was included in the results.

In order to evaluate the estimated impact of MenC vaccination on the population, the number of expected MDC cases with and without MenC vaccine intervention was predicted, using the regression models, for the period 2011-2013, both for Brazil and for each region. The impact was calculated as follows: Impact = (Ev - E)*100/E; where Ev = effect with vaccination for a given year, and E = effect without vaccination for the same year.

Analysis was performed using Microsoft Office Excel 2010 and STATA version 10.

*Ethical aspects* - This study was approved by the University of Brasília Faculty of Medicine Research Ethics Committee, Report No.908.096. The study used only non-nominal secondary data.

## RESULTS

**Fig. 1:**
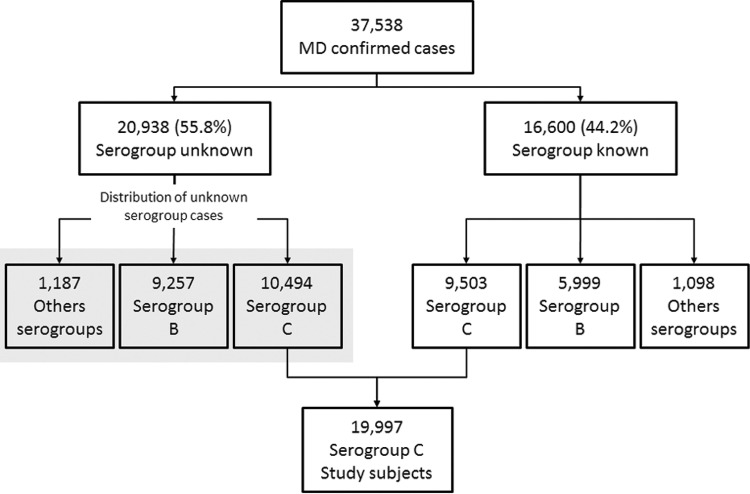
: description of meningococcal disease confirmed cases classified by serogroup (Brazil, 2001-2013).

A total of 37,538 MD cases were recorded in the Sinan database between 2001 and 2013. Among 16,600 (44.2%) confirmed MD cases, serogroup was known and distributed as follows: serogroup C, 9,503 (57.3%) cases; serogroup B, 5,999 (36.1%) cases; and other serogroups, 1,098 (6.6%) cases. In 20,938 (55.8%) MD confirmed cases serogroup was unknown. These unidentified serogroup cases were redistributed according to identified serogroups, by region of residence, age group, and trimester of symptom onset. Therefore, a total of 10,494 (50.1%) MD cases were attributed to serogroup C. Finally, a total of 19,997 (the sum of 9,503 and 10,494) patients with MDC were included in this study (Fig. 1).

**Fig. 2:**
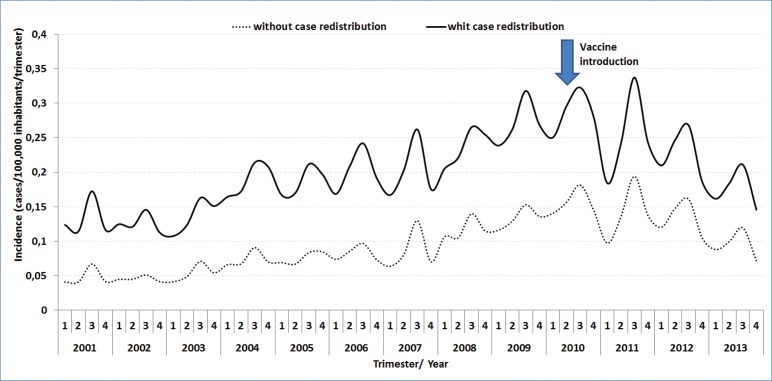
: meningococcal disease incidence with and without serogroup C redistribution per trimester and year of symptom onset (Brazil, 2001-2013).

Seasonal variation in the trimester incidence of MDC cases with (n = 19,997) and without (n = 9,503) redistribution of unknown serogroup was almost identical (Fig. 2).

Total MDC incidence between 2001 and 2009 (period before vaccine implementation) increased by approximately 100%, from 0.53 cases per 100,000 population in 2001 to 1.09 cases per 100,000 population in 2009. This increase occurred across all age groups and specifically, there was a 120% increase in the < 1 year age group (from 3.98 cases per 100,000 population in 2001 to 8.78 cases per 100,000 population in 2009) and a 72% increase in the 1-4 years age group (from 2.26 cases per 100,000 population in 2001 versus to 3.89 cases per 100,000 population in 2009) (Table I).

**TABLE I:** Serogroup C meningococcal disease incidence by age group and region of residence (Brazil, 2001-2013)

Region	Age group (years)	Meningococcal disease serogroup C incidence (cases/100,000)												

		2001	2002	2003	2004	2005	2006	2007	2008	2009	2010	2011*	2012*	2013*
	< 1	3.98	2.84	4.67	6.24	6.19	7.71	6.92	7.09	8.78	9.50	4.15	2.82	3.03
	1-4	2.26	1.85	2.18	2.77	2.96	3.13	2.78	3.40	3.89	3.68	3.16	1.70	0.89
Brazil	5-9	0.95	1.06	0.90	1.50	1.41	1.61	1.61	1.71	2.00	2.03	1.87	2.05	1.42
	≥ 10	0.23	0.25	0.26	0.37	0.36	0.38	0.43	0.54	0.62	0.71	0.70	0.71	0.58
	Total	0.53	0.50	0.54	0.76	0.75	0.81	0.81	0.95	1.09	1.15	1.01	0.91	0.70

	< 1	-	-	-	0.49	1.59	-	-	1.86	-	1.63	-	1.30	1.60
	1-4	0.48	0.68	0.27	0.33	0.20	1.21	0.54	-	0.92	0.48	0.70	0.42	0.22
North	5-9	0.18	0.70	0.69	1.16	-	0.23	1.06	0.22	0.56	-	0.22	0.42	0.22
	≥ 10	0.32	0.22	0.17	0.35	0.03	0.20	0.27	0.38	0.47	0.29	0.20	0.29	0.39
	Total	0.31	0.33	0.24	0.45	0.07	0.30	0.38	0.35	0.51	0.30	0.24	0.30	0.38

	< 1	0.00	0.26	1.69	1.47	2.09	3.14	2.37	2.62	4.90	3.11	2.64	1.32	2.55
	1-4	0.93	0.41	0.52	0.57	0.93	0.58	0.90	1.18	1.95	0.91	1.09	0.94	0.50
Northeast	5-9	0.63	0.80	0.41	0.32	0.68	0.71	0.87	0.64	1.45	1.06	1.01	1.19	0.53
	≥ 10	0.17	0.22	0.11	0.22	0.25	0.26	0.28	0.35	0.48	0.61	0.50	0.58	0.41
	Total	0.29	0.30	0.20	0.28	0.38	0.38	0.42	0.48	0.76	0.71	0.62	0.67	0.46

	< 1	4.40	0.00	3.02	4.88	5.66	7.22	8.05	7.64	9.08	4.53	2.65	2.39	1.49
	1-4	1.51	1.62	0.33	0.72	2.64	3.53	2.81	3.30	3.63	3.62	2.13	0.81	0.66
Midwest	5-9	0.42	0.20	0.37	0.74	0.18	0.55	0.98	0.59	1.38	1.21	1.10	0.88	0.41
	≥ 10	0.20	0.12	0.17	0.21	0.18	0.35	0.31	0.41	0.46	0.59	0.45	0.39	0.44
	Total	0.41	0.25	0.26	0.38	0.46	0.72	0.67	0.74	0.89	0.90	0.65	0.48	0.47

	< 1	8.48	6.59	9.17	12.10	11.06	14.93	13.72	12.86	15.65	18.97	7.62	5.16	4.19
	1-4	4.25	3.59	4.60	5.87	5.83	6.17	5.32	6.97	7.27	7.90	6.41	3.38	1.64
Southeast	5-9	1.75	1.85	1.56	2.75	2.86	2.75	2.79	3.48	3.56	3.96	3.61	4.10	3.09
	≥ 10	0.26	0.34	0.41	0.57	0.54	0.56	0.67	0.82	0.92	1.05	1.13	1.10	0.88
	Total	0.81	0.80	0.94	1.29	1.25	1.32	1.33	1.59	1.71	1.92	1.70	1.51	1.13

	< 1	2.24	1.51	2.77	5.00	5.26	3.42	2.05	4.55	4.20	4.85	1.32	0.44	2.79
	1-4	1.40	1.22	1.55	2.14	1.96	1.93	1.90	1.43	1.53	1.11	1.56	0.35	0.41
South	5-9	0.29	0.12	0.60	1.24	0.60	1.99	0.62	0.83	0.23	0.83	0.69	0.47	0.26
	≥ 10	0.20	0.14	0.18	0.16	0.25	0.19	0.17	0.22	0.19	0.19	0.19	0.17	0.18
	Total	0.32	0.24	0.35	0.46	0.47	0.50	0.34	0.40	0.32	0.35	0.32	0.20	0.23

*: years in bold indicate period pos-vaccine implementation.

MDC incidence increased in all geographical regions between 2001 and 2009. The highest increase occurred in the Northeast region (162%) (Table I). The Northern region was not included in this analysis because of the large number of trimesters with no cases or with all MD notified cases classified as unknown serogroup. The pattern in this region did not allow the redistribution of unknown serogroup MD cases for most of the study trimesters.

There was a historical trend of increased MDC across all age groups over the period 2001-2013, after controlling for seasonal trend and vaccine implementation (Table II). There were some exceptions: the Southeast region (1-4 years age group) and the Southern region (1-4 years, 5-9 years and > 10 years age groups). The model estimated an average annual increase of 0.14 cases per 100,000/trimester/year for the < 1 age group. This increase was greater in the Midwest and Southeast regions.

**TABLE II:** Association between serogroup C meningococcal disease incidence and selected variables* by age group (Brazil and regions, 2001-2013)

Variables	< 1 year			1-4 years			5-9 years			≥ 10 years		

	Coef.	CI 95%	p	Coef.	CI 95%	p	Coef.	CI 95%	p	Coef.	CI 95%	p
BRAZIL												
Vaccination: pre-implantation (2001-2009; reference)												
Implanted (2010)	-0.21	(-0.64, 0.22)	0.334	-0.09	(-0.20, 0.03)	0.136	0.01	(-0.09, 0.11)	0.873	0.02	(-0.003, 0.04)	0.094
Post-implantation (2011)	-1.13	(-1.59, -0.67)	0.000	-0.27	(-0.39, -0.15)	0.000	-0.06	(-0.17, 0.04)	0.251	-0.003	(-0.03, 0.02)	0.803
Post-implantation (2012)	-1.58	(-2.07, -1.09)	0.000	-0.64	(-0.77, -0.51)	0.000	-0.05	(-0.16, 0.06)	0.366	-0.01	(-0.04, 0.02)	0.448
Post-implantation (2013)	-1.49	(-2.00, -0.97)	0.000	-0.84	(-0.98, -0.70)	0.000	-0.22	(-0.34, -0.10)	0.000	-0.04	(-0.07, -0.005)	0.024
YEAR (2001-2013)	0.14	(0.09, 0.18)	0.000	0.05	(0.04, 0.07)	0.000	0.02	(0.01, 0.04)	0.000	0.01	(0.01, 0.01)	0.000
Trimester (January-March; reference)												
2 (April-June)	-0.10	(-0.35, 0.16)	0.457	0.02	(-0.03, 0.07)	0.430	0.01	(-0.04, 0.06)	0.718	0.01	(0.005, 0.02)	0.003
3 (July-September)	0.40	(0.14, 0.66)	0.003	0.15	(0.10, 0.21)	0.000	0.08	(0.03, 0.13)	0.002	0.04	(0.03, 0.05)	0.000
4 (October-December)	0.08	(-0.18, 0.33)	0.560	0.00	(-0.05, 0.06)	0.881	0.01	(-0.03, 0.06)	0.555	0.01	(0.004, 0.02)	0.005
Region of residence (Northeast; reference)												
Midwest	0.63	(0.30, 0.96)	0.000	0.30	(0.14, 0.46)	0.000	-0.03	(-0.09, 0.04)	0.411	0.00	(-0.02, 0.02)	0.769
Southeast	2.16	(1.87, 2.44)	0.000	1.10	(0.96, 1.25)	0.000	0.53	(0.46, 0.60)	0.000	0.09	(0.07, 0.11)	0.000
South	0.24	(-0.004, 0.48)	0.054	0.14	(0.02, 0.25)	0.018	-0.03	(-0.09, 0.03)	0.341	-0.04	(-0.05, -0.02)	0.000
NORTHEAST												
Pre-implantation (2001-2009; reference)												
Implanted (2010)	-0.31	(-0.63, 0.004)	0.053	-0.13	(-0.30, 0.03)	0.111	-0.01	(-0.15, 0.13)	0.881	0.05	(0.02, 0.08)	0.003
Post-implantation (2011-2013)	-0.82	(-1.10, -0.54)	0.000	-0.20	(-0.35, -0.05)	0.011	-0.06	(-0.19, 0.07)	0.351	0.00	(-0.02, 0.03)	0.763
YEAR (2001-2013)	0.12	(0.09, 0.15)	0.000	0.03	(0.01, 0.04)	0.003	0.02	(0.0004, 0.03)	0.044	0.01	(0.004, 0.01)	0.000
Trimester (January-March; reference)												
2 (April-June)	-0.14	(-0.44, 0.15)	0.340	-0.08	(-0.17, 0.01)	0.084	-0.03	(-0.10, 0.03)	0.341	0.00	(-0.01, 0.02)	0.636
3 (July-September)	0.13	(-0.11, 0.37)	0.283	-0.03	(-0.13, 0.06)	0.477	0.00	(-0.08, 0.07)	0.930	0.02	(0.01, 0.04)	0.006
4 (October-December)	0.10	(-0.19, 0.40)	0.486	-0.08	(-0.17, 0.02)	0.110	-0.03	(-0.09, 0.04)	0.426	0.00	(-0.01, 0.02)	0.694
MIDWEST												
Pre-implantation (2001-2009; reference)												
Implanted (2010)	-1.27	(-2.29, -0.24)	0.016	-0.05	(-0.47, 0.37)	0.801	0.04	(-0.14, 0.22)	0.632	0.03	(-0.02, 0.08)	0.241
Post-implantation (2011-2013)	-2.33	(3.24, -1.43)	0.000	-0.82	(-1.20, -0.43)	0.000	-0.10	(-0.27, 0.06)	0.216	-0.03	(-0.08, 0.02)	0.265
YEAR (2001-2013)	0.21	(0.11, 0.32)	0.000	0.08	(0.04, 0.12)	0.000	0.02	(0.003, 0.04)	0.023	0.01	(0.004, 0.02)	0.001
Trimester (January-March; reference)												
2 (April-June)	0.20	(-0.61, 1.01)	0.628	-0.09	(-0.35, 0.17)	0.499	-0.05	(-0.16, 0.06)	0.398	0.01	(-0.02, 0.03)	0.711
3 (July-September)	0.11	(-0.62, 0.84)	0.759	0.22	(-0.05, 0.48)	0.104	0.05	(-0.07, 0.16)	0.423	0.04	(0.01, 0.07)	0.014
4 (October-December)	-0.23	(-1.04, 0.57)	0.571	-0.12	(-0.38, 0.15)	0.385	-0.04	(-0.16, 0.07)	0.451	0.00	(-0.03, 0.03)	0.894
SOUTHEAST												
Pre-implantation (2001-2009; reference)												
Implanted (2010)	0.70	(-0.16, 1.56)	0.108	-0.04	(-0.53, 0.46)	0.883	0.08	(-0.13, 0.28)	0.469	0.02	(-0.03, 0.07)	0.374
Post-implantation (2011-2013)	-3.08	(-3.87, -2.29)	0.000	-0.86	(-1.44, -0.27)	0.004	-0.15	(-0.35, 0.05)	0.154	-0.02	(-0.08, 0.03)	0.424
YEAR (2001-2013)	0.23	(0.14, 0.32)	0.000	0.06	(-0.01, 0.13)	0.096	0.06	(0.03, 0.08)	0.000	0.02	(0.01, 0.02)	0.000
Trimester (January-March; reference)												
2 (April-June)	-0.19	(-0.69, 0.30)	0.451	0.27	(0.11, 0.42)	0.001	0.15	(0.06, 0.25)	0.002	0.05	(0.03, 0.06)	0.000
3 (July-September)	0.88	(0.37, 1.40)	0.001	0.44	(0.27, 0.62)	0.000	0.30	(0.19, 0.40)	0.000	0.08	(0.06, 0.10)	0.000
4 (October-December)	0.04	(-0.45, 0.54)	0.865	0.15	(-0.01, 0.31)	0.059	0.15	(0.06, 0.25)	0.002	0.04	(0.02, 0.06)	0.000
SOUTH												
Pre-implantation (2001-2009; reference)												
Implanted (2010)	0.05	(-0.52, 0.62)	0.863	-0.15	(-0.42, 0.12)	0.274	0.01	(-0.19, 0.21)	0.945	0.00	(-0.03, 0.02)	0.718
Post-implantation (2011-2013)	-0.96	(-1.46, -0.45)	0.000	-0.24	(-0.48, 0.00)	0.052	-0.11	(-0.29, 0.08)	0.269	-0.01	(-0.03, 0.01)	0.514
YEAR (2001-2013)	0.06	(0.00, 0.12)	0.036	0.00	(-0.03, 0.03)	0.876	0.01	(-0.01, 0.03)	0.517	0.00	(-0.002, 0.003)	0.509
Trimester (January-March; reference)												
2 (April-June)	-0.02	(-0.57, 0.52)	0.930	0.03	(-0.17, 0.22)	0.798	0.05	(-0.05, 0.15)	0.360	0.01	(-0.01, 0.02)	0.355
3 (July-September)	0.77	(0.34, 1.21)	0.000	0.14	(-0.05, 0.32)	0.140	0.13	(0.02, 0.24)	0.023	0.02	(0.01, 0.04)	0.002
4 (October-December)	0.21	(-0.33, 0.75)	0.452	0.16	(-0.04, 0.35)	0.113	0.06	(-0.04, 0.16)	0.258	0.01	(-0.01, 0.03)	0.173

*: associations were estimated based on generalised least square (GLS) regression models considering as dependent variable the trimester incidence rates for each year. Final models fail to meet the linearity assumption for ages 1-4 in Southeast region, 5-9 in Brazil and ≥ 10 in Brazil and Southeast; and the independence assumption for ages 1-4 in Brazil and Southeast and 5-9 and ≥ 10 in Brazil; CI: confidence interval.

A statistically significant increase in MDC was found in the months of July and September in all age groups across Brazil, after controlling for historical trends and vaccine implementation (Table II).

**Fig. 3:**
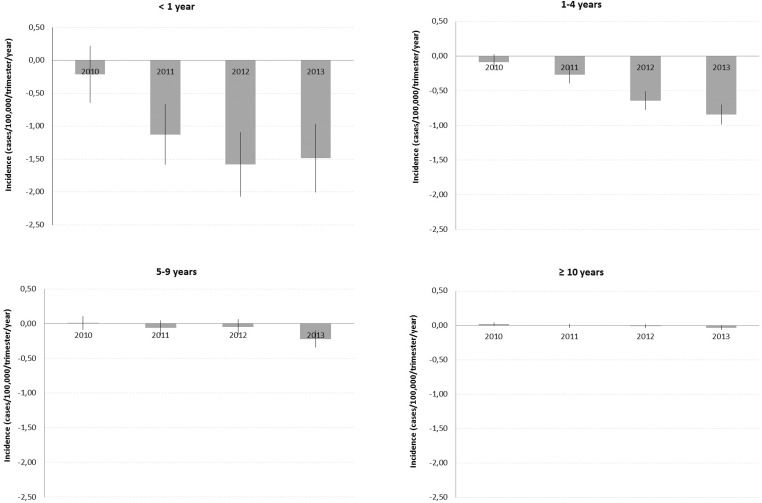
: average reduction of serogroup C meningococcal disease incidence * by age group after vaccine implementation (Brazil, 2010-2013); *: average reductions were estimated based on generalised least square (GLS) regression models considering as dependent variable the trimester incidence rates for each year, and adjusted by historical trend and seasonality.

The effect of vaccination on the average trimestral MDC incidence was adjusted according to historical trends, seasonal trends, and geographical regions. In the years following vaccine implementation (2011, 2012, and 2013), increasing gradients in the reduction of the incidence rates were observed across all age groups. Conversely, the vaccination effect decreased with age of the study population (Table II, Fig. 3).

A decrease in mean MDC incidence was found in subjects aged < 1 year after the introduction of the MenC vaccine, as follows: -1.13 cases/100,000/trimester/year in 2011, -1.58 in 2012, and -1.49 in 2013 (p < 0.001). A decrease in mean MDC incidence was also found in the 1-4 years age group: -0.27 cases/100,000/trimester/year in 2011, -0.64 in 2012, and -0.84 in 2013 (p < 0.001). In the 5-9 years and ≥ 10 years age groups a decrease in MDC incidence was observed only in 2013: -0.22 cases/100,000/trimester/year (p < 0.001) and -0.04 cases/100,000/trimester/year (p = 0.024), respectively (Table II, Fig. 3).

The four regions analysed showed a statistically significant MDC incidence reduction in the < 1 year and 1-4 years age groups (not including the Southern region). The largest impact was found in the Southeast and Midwest regions: -3.08 and -2.33 cases/100,000/trimester/year, respectively, in the < 1 year group, and -0.86 and -0.82 cases/100,000/trimester/year, respectively, in the 1-4 years age group. Smaller reductions were observed in the Northeast and Southern regions: -0.82 and -0.96 cases/100,000/trimester/year, respectively, in the < 1 year group, and -0.20 and -0.24 cases/100,000/trimester/year, respectively, in the 1-4 years age group (Table II).

Estimates using the same time series models but considering MDC incidence without the redistribution of unidentified serogroup cases produced almost identical results. However, they systematically underestimated the coefficient.

Table III shows the results of the impact of MenC vaccination in terms of percentage reduction in MDC cases, as derived from the regression models. For children aged < 1 and 1-4 years old, the reduction in the number of cases in Brazil, over the period 2011-2013, was 65.7% and 51.8%, respectively. The reduction, per region, among children aged < 1 year was highest in the Midwest region, and smallest in the Northeast region (Table III).

**TABLE III:** Percentage case reduction estimated by the regression model for children aged < 1 year and children aged 1-4 years in Brazil and regions in the years following the introduction of meningococcal C vaccination

Brazil	< 1 year		1-4 years	
	
	Variation in MDC cases (%)	CI 95%	Variation in MDC cases (%)	CI 95%
2010	1.5	(119.6, 22.5)	-4.2	(-23.2, 14.8)
2011	-54.6	(-75.3, -33.8)	-21.8	(-40.8, -2.8)
2012	-70.9	(-91.7, -50.2)	-57.6	(-76.8, -38.4)
2013	-70.7	(-91.6, -49.8)	-74.5	(-92.7, -56.2)
2011-2013	-65.7	(-86.5, -44.9)	-51.8	(-70.6, -33.0)
Northeast				
2010	-28.2	(-52.5, -3.9)	-44.8	(-90.9, 1.2)
2011-2013	-56.7	(-76.0, -37.4)	-55.0	(-97.2, -12.9)
Midwest				
2010	-55.0	(-97.0, -13.1)	-9.4	(-46.5, 27.8)
2011-2013	-83.7	(-100.0, -51.1)	-73.4	(-100.0, -39.1)
Southeast				
2010	13.5	(-2.7, 29.7)	4.5	(-6.8, 15.8)
2011-2013	-65.4	(-82.2, -12.2)	-42.9	(-72.3, -13.5)
South				
2010	4.2	(-34.7, 43.0)	-32.6	(-75.5, 10.3)
2011-2013	-60.1	(-91.7, -28.5)	-44.0	(-88.4, 0.4)

CI: confidence interval; MDC: meningococcal disease serogroup C.

## DISCUSSION

This was the first study to evaluate the impact of the introduction of MenC vaccination in Brazil, taking into account national data as well as regional and age differentials.

The estimation of vaccination impact included adjustments for time and seasonal trends, as a failure to take these into account could have affected the validity of the vaccination impact estimates. Overall, there was a historical trend of increased MDC across all age groups over the period 2001-2013, particularly in the Midwest and Southeast regions. The study estimated that the mean increase was 0.14 cases/100,000/trimester/year in the < 1 year age group. These findings reflect the appearance of a serogroup C strain belonging to a hypervirulent lineage (ST-103), first described in São Paulo, in the Southeast region in the early 2000s (Lemos et al. 2007). This strain subsequently spread to other regions (Cardoso et al. 2012, Tauil et al. 2014). Furthermore, a seasonal predominance of the disease was observed in the winter months, from July to September. This is a known characteristic of MD in Brazil and also occurs in other countries (Halperin et al. 2012).

The MDC incidence reduction in Brazil was consistent with MenC vaccination implementation. A significant reduction in the number of estimated cases following the introduction of the vaccination program (2011, 2012 and 2013) was observed in children aged < 1 year and 1-4 years. A statistically significant decrease in the MDC incidence after the introduction of the vaccination program was also found in the 5-9 years and ≥ 10 years age groups in 2013, although the limited effects should be interpreted with caution. Furthermore, this study identified geographical region as a MenC vaccine impact modifying variable in Brazil. Earlier and greater impacts were found in the Midwest region and in the Southeast region.

The MDC reduction in Brazil in children aged < 1 year between 2011 and 2013 [65.7%; 95% confidence interval (CI): 44.9-86.5%] shows the effect (direct and indirect) of vaccination on the target population. In the United Kingdom and in Spain, disease incidence in children aged < 1 year following MenC vaccination decrease by 77.5% and 85%, respectively (Balmer et al. 2002, Larrauri et al. 2005).

The reduction in estimated cases of MDC in children aged 1-4 years across Brazil, due to the implementation of vaccination program, was 51.8% (95%CI: 33.0-70.6%). This was smaller than the reduction estimated for children in the < 1 year age group and can be explained by the fact that this group includes children who may not have been vaccinated. The MDC incidence reduction attributed to vaccination in the 1-4 year age group in other countries was approximately 85% (Balmer et al. 2002, Larrauri et al. 2005).

The limited impact during 2013 in children aged 5-9 years (p < 0.001) and ≥ 10 years (p = 0.024) may be a consequence of shifts in the cohort of vaccinated children over time, and particularly among children aged 5-6 years. The hypothesis of MD cyclical trends, dependent on natural immunity, and the occurrence of herd immunity should also be addressed. It is not clear whether younger vaccinating children will have an impact, albeit low, on meningococcal carriage and whether this will have any indirect impact on disease incidence among older children. It is important to note that the prevalence of meningococcal carriage described in a meta-analysis study (Christensen et al. 2010) was found to be higher in adolescence and early adulthood (peak of 23.7% at 19 years of age) than in childhood (4.5% among infants and 7.7% among 10-year-old subjects). Studies assessing these hypotheses are required.

Overall, the results of this study were comparable to those described in the literature (Balmer et al. 2002, Larrauri et al. 2005, Bettinger et al. 2009). The small differences between countries in the estimated impact of vaccination strategies can be justified, at least in part, by the methodology used for analysis, the specificities of disease epidemiology, and differences in vaccine implementation in each country. It is important to highlight that the impact estimate is derived from an ecological study, in which children in the pre-vaccination period were compared with children in the post-vaccination period; however, children in the post-vaccination period may or may not have been vaccinated, may have been vaccinated according to different vaccination schedules, or may have received different vaccine doses. Moreover, the model chosen for estimating the impact of vaccination was an adjusted time series; therefore, estimated impact parameters will be more conservative than those obtained using unadjusted analysis.

With regards to the age groups targeted by vaccination programs, the objective in Brazil was to directly immunise children aged < 2 years, specifically unweaned infants, as this age group has the highest at risk of meningococcal disease in the country. Unlike other countries, in Brazil the incidence of MD does not peak among adolescents and young adults. This only occurs during outbreaks when high rates are found among adolescents and adults (Cardoso et al. 2012, Gorla et al. 2012, Iser et al. 2012). Almost all countries (Trotter et al. 2004, Trotter & Ramsay 2007, Bettinger et al. 2009) implementing MenC vaccination in their immunisation programmes have opted to introduce catch-up campaigns for adolescents, and these appear to have been crucial for generating high levels of herd immunity (Ramsay et al. 2003, Kinlin et al. 2009). Conjugate vaccines have the ability to reduce the prevalence of meningococcal carriage, resulting in a reduced risk of the disease, even among individuals who have not been vaccinated (Maiden et al. 2008, Campbell et al. 2010). On the basis of this study’s data, it was not possible to find a relevant MDC reduction in other age groups not targeted by vaccination following the introduction of the program. An example of this is no reduction in MDC cases among children aged < 3 months after the introduction of vaccination programs in Brazil (de Moraes 2016). In the United Kingdom, there was a reduction in the total number of serogroup C cases in children < 3 months, attributed to an indirect protective effect of the vaccine (Campbell et al. 2009). These findings should be considered when evaluating the inclusion of other age groups for vaccination in Brazil.

Important sub-national variations, taking into account the regional analysis of the MenC vaccination impact, are described. Brazil is country with important variations in the implementation of health policies, such as immunisation programs, as a consequence of regional development and a decentralised health system. Ignoring this diversity may lead to misleading results. The sub-national analysis was useful to formulate hypotheses that may explain the lower than anticipated vaccine impact in some Brazilian regions. These hypotheses may address differentials related to vaccine coverage, quality of the disease surveillance, as well as disease burden influenced by population density and presence of urban clusters.

Some of the study results (increasing impact of vaccination over time and regional variations in vaccination impact) may be explained, at least in part, by variations in the vaccination coverage and disease burden. MenC vaccination implementation in Brazil took place between March and November 2010, and high coverage was achieved in the months following implementation in almost all the country’s regions. The only exception was the Northeast region, where the target recommended by the PIN-MS (95% coverage) was only reached in 2013 (SI-PNI/MS). This may explain the low impact of the intervention in this region. The Southeast region consistently had the highest MDC incidence, both prior to and following the introduction of the vaccination program. Furthermore, there was a strong trend of increasing MDC incidence, in the Southeast region, as well as in the Midwest region, in the period before the vaccination implementation. This may have influenced the high impact of the implementation of vaccination in these regions. The limited median impact found in the Southern region may be related to the low MDC incidence and the late increase in serogroup C circulation between 2012 and 2013, which are specific to these regions.

The results of the study must be interpreted with caution, taking into account a number of limitations. First, this study used secondary data sources; therefore, the possibility of underreporting and inconsistency in recording the variables of interest and case classification should be considered. Nevertheless, studies have shown that the Sinan system data is valid in the context of changes in the historical trends of the disease (Azevedo et al. 2013). Secondly, the database used in this study contained a high percentage of MD cases with unidentified serogroups. This limitation was minimised by the proportional redistribution of these cases to achieve the same distribution as the laboratory confirmed cases, by region of residence, age group, and trimester of symptom onset (Miller et al. 2002). Serogroup identification improved over the study period; if the cases of unidentified serogroup had not been redistributed and had been excluded from the analysis, this may have resulted in a significant underestimation of the incidence, increased random error in the estimates (and the range of the confidence intervals), and selection bias. A further limitation is the GLS regression assumptions. Violation of linearity and independence assumptions were identified in few analyses and their results must be considered with caution. Finally, the fact that this is an ecological study limits the generalisability of results, for example, these findings cannot be compared with those of individual-based studies.

In conclusion, vaccination against MDC in Brazil is likely to have an impact on the target population (children aged < 1 year) for all analysed regions, to a variable extent, and on the 1-4-year- old cohort. Nevertheless, it is our view that there is scope for improving the vaccination strategy adopted in Brazil. Vaccinating other age groups, for example adolescents, may increase the MenC vaccination impact, including a possible indirect effect on non-vaccinated groups. Moreover, our study results highlight the need to develop investigations to improve our understanding of how regional differences affect vaccine impact. Furthermore, monitoring vaccine coverage and further development of the MD surveillance system are fundamental to improve vaccine impact and to decrease the disease burden in Brazil.
